# Erythrocytic Mobilization Enhanced by the Granulocyte Colony-Stimulating Factor Is Associated with Reduced Anthrax-Lethal-Toxin-Induced Mortality in Mice

**DOI:** 10.1371/journal.pone.0111149

**Published:** 2014-11-10

**Authors:** Hsin-Hou Chang, Ya-Wen Chiang, Ting-Kai Lin, Guan-Ling Lin, You-Yen Lin, Jyh-Hwa Kau, Hsin-Hsien Huang, Hui-Ling Hsu, Jen-Hung Wang, Der-Shan Sun

**Affiliations:** 1 Department of Molecular Biology and Human Genetics, Tzu-Chi University, Hualien, Taiwan; 2 Institute of Medical Sciences, Tzu-Chi University, Hualien, Taiwan; 3 Department of Microbiology and Immunology, National Defense Medical Center, Taipei, Taiwan; 4 Institute of Preventive Medicine, National Defense Medical Center, Taipei, Taiwan; 5 Department of Medical Research, Tzu Chi General Hospital, Hualien, Taiwan; University of Pittsburgh, United States of America

## Abstract

Anthrax lethal toxin (LT), one of the primary virulence factors of *Bacillus anthracis*, causes anthrax-like symptoms and death in animals. Experiments have indicated that levels of erythrocytopenia and hypoxic stress are associated with disease severity after administering LT. In this study, the granulocyte colony-stimulating factor (G-CSF) was used as a therapeutic agent to ameliorate anthrax-LT- and spore-induced mortality in C57BL/6J mice. We demonstrated that G-CSF promoted the mobilization of mature erythrocytes to peripheral blood, resulting in a significantly faster recovery from erythrocytopenia. In addition, combined treatment using G-CSF and erythropoietin tended to ameliorate *B. anthracis*-spore-elicited mortality in mice. Although specific treatments against LT-mediated pathogenesis remain elusive, these results may be useful in developing feasible strategies to treat anthrax.

## Introduction

Infection with *Bacillus anthracis*, a gram-positive spore-forming bacterium, can lead to life-threatening anthrax [1]. Anthrax lethal toxin (LT) is comprised of a protective antigen (PA, 83 kDa) and lethal factor (LF, 90 kDa) [2]–[4], and is one of the primary virulence factors of *B. anthracis*. LF is a specific metalloprotease for mitogen-activated protein kinase (MAPK) kinases (MKKs/MEKs) [5], and can thus disrupt MAPK signaling cascades including p38 MAPK, p42/44 extracellular signal-regulated kinase (ERK), and c-Jun N-terminal kinase (JNK) [6], [7]. All of these 3 MAPK pathways are critical in maintaining fundamental cellular homeostasis, including cell proliferation, differentiation, and apoptosis [8]. LF can be delivered into cells by PA, a cell-receptor binding component [3], [9]. Although experimental LT treatments may not reproduce the full pathogenesis of anthrax, LT studies in cell or animal models have revealed certain pathogenic progressions. Various cell types, which include macrophages [10], [11], dendritic cells [12], lymphocytes [13], [14], erythrocytes [15], and megakaryocytes [16], cardiomyocytes [17], and smooth muscle [17], are sensitive to LT treatment. LT has been shown to suppress the differentiation and maturation of the progenitors of macrophages, megakaryocytes, and erythrocytes [15], [16], [18]. In addition, blood cell count analyses have indicated that LT treatment significantly reduced levels of circulating red blood cells (RBCs) and platelets in mice [15], [16], suggesting multiple targets of LT in hematopoietic lineage cells. Deficiencies of platelets and RBCs may lead to hemorrhage and lethal hypoxic damage [19], [20]. Because high levels of LT accumulate in the body when anthrax enters the bacteremia stage, death is typically inevitable even after aggressive antibiotic treatments. This suggests that a specific treatment to overcome the toxic effect is crucial in controlling the disease [21]. Unfortunately, an effective therapeutic approach against LT remains elusive.

Cytokine treatments, particularly hematopoietic growth factors, have been used in various clinical settings to rescue pathological defects [22]. Our previous demonstration was the first to indicate that thrombopoietin (TPO), a megakaryopoiesis-enhancing cytokine [23], can ameliorate LT-induced thrombopoiesis suppression, thrombocytopenia, and likely reduce the mortality in mice [16]. Our data also revealed that erythropoietin (EPO), a potent erythropoiesis-stimulating cytokine [24], ameliorated LT-induced erythropoiesis suppression (particularly those precursors in early erythropoiesis stages), erythrocytopenia, and reduced mortality rates from 100% to 50% after lethal-dose LT challenges in experimental mice [15]. Bone marrow is the primary stem niche supporting erythropoiesis, displaying technically-divided 4-differentiation stages of erythroblasts based on the expression levels of surface markers CD71 and TER-119 [25]. The transferrin receptor (CD71) is first expressed on early erythroblasts, such as erythroid burst-forming units (BFU-Es) and erythroid colony-forming units (CFU-Es) cells. Erythrocytic CD71 is downregulated by more mature erythroblasts [26]. By contrast, TER-119 is primarily expressed on relatively mature erythroblasts, reticulocytes, and mature erythrocytes [27]. Accordingly, 4 cell populations (CD71^high^TER-119^med^, CD71^high^TER-119^high^, CD71^med^TER-119^high^, and CD71^low^TER-119^high^) can be defined, which are morphologically equivalent to proerythroblasts (flow cytometry-gated region 1; R1), basophilic erythroblasts (R2), late basophilic and polychromatophilic erythroblasts (R3), and orthochromatophilic erythroblasts (R4), from the early to late stages of erythroid differentiation, respectively [25]. Following these approaches, we are thus able to characterize the mechanism to use hematopoietic cytokines/growth factors as ameliorative agents to rescue anthrax LT-induced mortality [15], [16].

The granulocyte colony-stimulating factor (G-CSF) has been found to regulate granulopoiesis [28], and is a multifunctional cytokine. For example, it has been found to stimulate cell proliferation, differentiation, enhance hematopoiesis, mobilize hematopoietic stem cells, and induce anti-apoptotic and anti-inflammatory effects [29]–[31]. Both G-CSF and EPO are U.S. FDA-approved drugs. Although the mechanism remains unclear, combined treatments using G-CSF and EPO were shown to ameliorate aplastic anemia in patients with myelodysplastic syndrome [32]–[36]. Given that EPO treatments are beneficial for LT-challenged mice [15], we hypothesized that combining G-CSF and EPO may be useful in treating anthrax. Consequently, we used mouse models to discuss the combined treatments of G-CSF and EPO on reduced anthrax LT and spore-induced mortality. In addition, we also discussed the differential erythropoietic regulation in response to G-CSF and EPO treatments.

## Materials and Methods

### Ethics Statement

Our research approaches involving experimental mice were approved by the Institutional Animal Care and Use Committee of Tzu Chi University (Approval ID: 98104) and the National Defense Medical Center (Approval ID: AN-100-04).

### Toxins and spores

*B. anthracis*-derived LT was purified according to previously described procedures [49]. LT was delivered in a 1:5 ratio of LF and PA [16]. Spores derived from the *B. anthracis-*nonencapsulated mutant strain (pXO1^+^, pXO2^−^) were purchased from the American Type Culture Collection (Manassas, VA, USA) (ATCC 14186).

### Erythroid colony-forming cell assay

The erythroid colony-forming cell assay was conducted according to the manufacturer’s instructions (MethoCult M3334, StemCell Technologies). For the *in* *vitro* erythroid colony-forming cell assay, bone marrow cells were collected from the femurs and tibiae of C57BL/6J mice. C57BL/6J mice (males, 8–10 wk of age) were obtained from the National Laboratory Animal Center (Taipei, Taiwan) and kept in a specific pathogen-free (SPF) environment in the experimental animal center of Tzu Chi University. For the *ex* *vivo* erythroid colony-forming cell assay, C57BL/6J mice were retro-orbitally injected with 55 µg/kg/d of recombinant human G-CSF (Filgrastim, Kirin, Tokyo, Japan) in 250 µl saline, once daily for 5 d, initiated 5 d before the challenges of a lethal dose of LT (1.5 mg/kg in 250 µl saline, retro-orbitally injected). Treatments using saline, G-CSF, and LT alone were used as comparison controls. Bone marrow cells were collected at 69 h after LT treatment and flushed with Roswell Park Memorial Institute medium (RPMI)-1640 containing 20% anticoagulant acid citrate dextrose formula A (ACD-A: 38 mM citric acid, 75 mM trisodium citrate, 139 mM D-glucose, 12.5 mM EDTA [15]). After depleting RBCs by adding a hypotonic buffer (153 mM NH_4_Cl and 17 mM Tris-HCl) at room temperature for 10 min, 100 µl of remaining cells (9×10^5^/ml) were resuspended in Iscove’s Modified Dulbecco’s Medium (IMDM) (StemCell Technologies) and mixed with 1 ml of semisolid methylcellulose-based medium containing 3 units of EPO. Finally, each 1.1 ml of methylcellulose-cell suspension was mixed with or without a dose of G-CSF (20 ng/ml or 764 ng/ml) and duplicate seeded in 35-mm dishes. A G-CSF dose of 20 ng/ml was used in the colony-forming cell assay for hematopoietic cells [50]. Because the volume of mice blood is 70–80 ml/kg [51], a dose of 764 ng/ml approximated the dose used to ameliorate LT-induced mortality in the experiments. Two doses of G-CSF (20 ng/ml and 764 ng/ml) were added to the medium supplement of the erythroid colony-forming cell assay. The cultures were incubated at 37°C for 14 d. Dynamic changes in colony number were measured on Days 3, 7, and 14 after initiating the colony assay. The erythroid colonies were separated into 3 groups by size: small (8–50 cells), medium (more than 50, but less than 200 cells), and large (more than 200 cells).

### Analysis of erythropoiesis in bone marrow

Bone marrow cells were purified and blocked with 5% bovine serum albumin in RPMI medium at 37°C for 1 h and incubated in 500 µl RPMI-1640 medium with 1 µl of fluorescein (FITC)-conjugated rat anti-mouse CD71 antibody (BioLegend) and 3 µl of R-Phycoerythrin (R-PE)-conjugated rat anti-mouse TER-119 antibody (BD Immunocytometry System) at 37°C for 1 h. After washing with phosphate-buffered saline (PBS), the cells were measured and analyzed using a FACSCalibur flow cytometer and the CellQuest™ Pro program (Becton-Dickinson).

### Flow cytometry analysis of peripheral blood cells of G-CSF-treated EGFP mice

EGFP mice [C57BL/6J-Tg (Pgk1-EGFP) 03Narl, males, 10–12 wk of age] were obtained from the National Laboratory Animal Center (Taipei, Taiwan) and maintained in the aforementioned SPF environments. EGFP mice were retro-orbitally injected with G-CSF (55 µg/kg/d in 250 µl saline) once daily for 4 d. To detect the erythrocytes’ specific surface markers, 50 µl of retro-orbital blood samples were obtained 22, 44, 66, and 94 h after the initial G-CSF injection, and subsequently mixed with 450 µl of anticoagulant ACD-A (1:9). Cells were incubated in 300 µl of RPMI-1640 medium with 3 µl of the R-Phycoerythrin (R-PE)-conjugated rat anti-mouse TER-119 antibody (BD Immunocytometry System) at 37°C for 1 h. After washing with PBS, the cells were analyzed using a FACSCalibur flow cytometer and the CellQuest™ Pro program.

### G-CSF treatment to reduce LT-induced mortality

C57BL/6J mice (males, 8–10 wk of age) were retro-orbitally injected with 55 µg/kg/d of recombinant human G-CSF in 250 µl saline, once daily for 5 d, initiated 5 d before or 1 d after the challenges of a lethal dose of LT (1.5 mg/kg in 250 µl saline, retro-orbitally injected). Treatments using saline, G-CSF, and LT alone were used as comparison controls. Because no suitable potential predictor of death/survival exists for LT-challenged mice, we used death as an endpoint for the survival experiment. The survival time and mortality of mice were recorded after the LT challenges. LT treatment in mice did not induce obvious discomfort and body weight loss, except for reducing activities. The experimental mice were continually monitored up to 250 h for every 4–6 h. All surviving mice were monitored each day for 2 subsequent mo. For hematopoietic parameters, 50 µl of retro-orbital blood samples were collected at 22, 44, and 66 h after LT challenges and analyzed by an automated hematology analyzer (KX-21, Sysmex Corporation).

### Combined treatments with G-CSF and EPO to reduce anthrax-spore-induced mortality

C57BL/6J mice were retro-orbitally injected with G-CSF (55 µg/kg/d in 250 µl saline) daily for 5 consecutive d or injected with a combination of recombinant human EPO (rhEPO, Neorecormon, Roche, Mannheim, Germany) (2 IU/g, in 250 µl saline) twice at 24 and 48 h after injecting spores (1×10^7^ in 1 ml saline, intraperitoneal injection). The survival times and mortality of mice were recorded. Because no suitable potential predictor of death/survival exists for spore-challenged mice, we used death as an endpoint for the survival experiment. Spore treatment in mice did not induce obvious discomfort and body weight loss, except for reducing activities. The experimental mice were continually monitored up to 15 d for every 4–6 h. All surviving mice were monitored each day for 2 subsequent mo.

### Statistics

All results are presented as the mean ± SD (standard deviation) for each group. Data significance was examined by one-way ANOVA followed by the post-hoc Bonferroni-corrected *t*-test. Univariate Kaplan-Meier analysis was used to compare the difference in survival rate between groups with various treatments. *P*-values were calculated and log-rank tests were performed to determine statistical significance. A probability of type 1 error α = 0.05 was recognized as the threshold of statistical significance. Statistical analysis was conducted using the statistical software SPSS, version 17.0 (SPSS Inc., Chicago, IL, USA).

## Results

### G-CSF treatment promoted erythrocytic differentiation and proliferation *in* *vitro* and *ex* *vivo*

**Figure 1:**
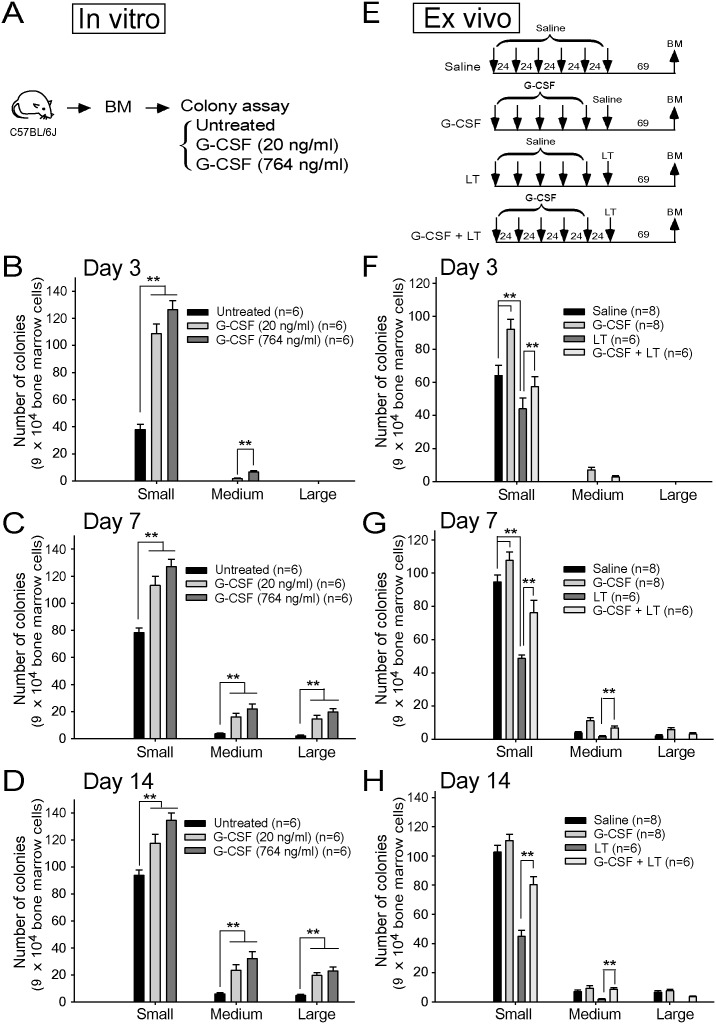
Erythroid colony-forming cell assays to measure the effect of G-CSF on erythropoiesis. The experimental outlines of *in* *vitro* (A) and *ex* *vivo* (E) analyses are shown. An *in* *vitro* assay was performed using murine bone marrow (BM) cells that were incubated with [20 ng/ml (n = 6) or 764 ng/ml (n = 6)], or without G-CSF (n = 6). The colonies were quantified on Days 3 (B), 7 (C), and 14 (D). Untreated bone marrow cells were used as a control. Colony numbers of bone marrow cells from mice, which were treated with G-CSF (n = 8), LT (n = 6), or G-CSF and LT (n = 6), were measured on Days 3 (F), 7 (G), and 14 (H) following the *ex* *vivo* colony assay. Bone marrow cells from saline treated mice (n = 8) served as controls. **P<0.01 was compared between the indicated groups. Data are shown as mean ± standard deviation (SD) and represent results from 2 independent experiments. The mouse drawing used in this and all following figures was originally published in *Blood.* Huang, H. S., Sun, D. S., Lien, T. S. and Chang, H. H. Dendritic cells modulate platelet activity in IVIg-mediated amelioration of ITP in mice. *Blood*. 2010; 116: 5002–5009. © the American Society of Hematology.

To elucidate the role of G-CSF on erythrocytic differentiation and proliferation, an *in* *vitro* erythroid colony-forming cell assay was performed to quantify BFU-Es and CFU-Es. Control groups without using filgrastim G-CSF supplements formed only medium-sized colonies by Day 7 (Figure 1C). By contrast, G-CSF treatments accelerated the formation of medium-sized colonies, which appeared earlier by Day 3 (Figure 1B). Following G-CSF treatment, the numbers of colonies in all colony sizes were greater than those in the untreated control groups (Figure 1B–1D). Based on the traditional concept that G-CSF primarily regulates granulopoiesis [28], those colonies to be affected by G-CSF treatment may not be exclusively erythroid-origin cells. Consequently, 3, 3′-diaminobenzidine tetrahydrochloride (DAB) [37] was used to identify erythroid colonies, and the pseudoperoxidase activity of erythroid cells was stained on Day 14. Compared with the untreated groups, the number of DAB^+^ colonies was greater in G-CSF-treated groups (Figure S1). This data indicated that G-CSF treatment enhances the proliferation and differentiation of erythrocytes *in* *vitro*. Further experiments were performed to investigate the effect of G-CSF and LT treatments *ex* *vivo* (Figure 1E). The number of erythroid colonies sharply decreased with LT treatment on Days 3, 7 and 14 (Figure 1F–1H). Medium-sized colonies first appeared on Day 3 in G-CSF treated groups (Figure 1F), compared with saline and LT-treated groups, whereas medium-sized colonies only appeared on Day 7 (Figure 1G). In addition, G-CSF treatments ameliorated LT-induced suppression on erythropoiesis in the *ex* *vivo* erythroid colony-forming cell assay (Figure 1F–1H). These results indicated that G-CSF promoted erythrocytic proliferation and differentiation *in* *vitro* and *ex* *vivo* and ameliorated LT-induced erythropoiesis suppression in the *ex* *vivo* erythroid colony-forming cell assay.

### G-CSF treatment promoted mobilization of newly synthesized RBC to peripheral blood

**Figure 2:**
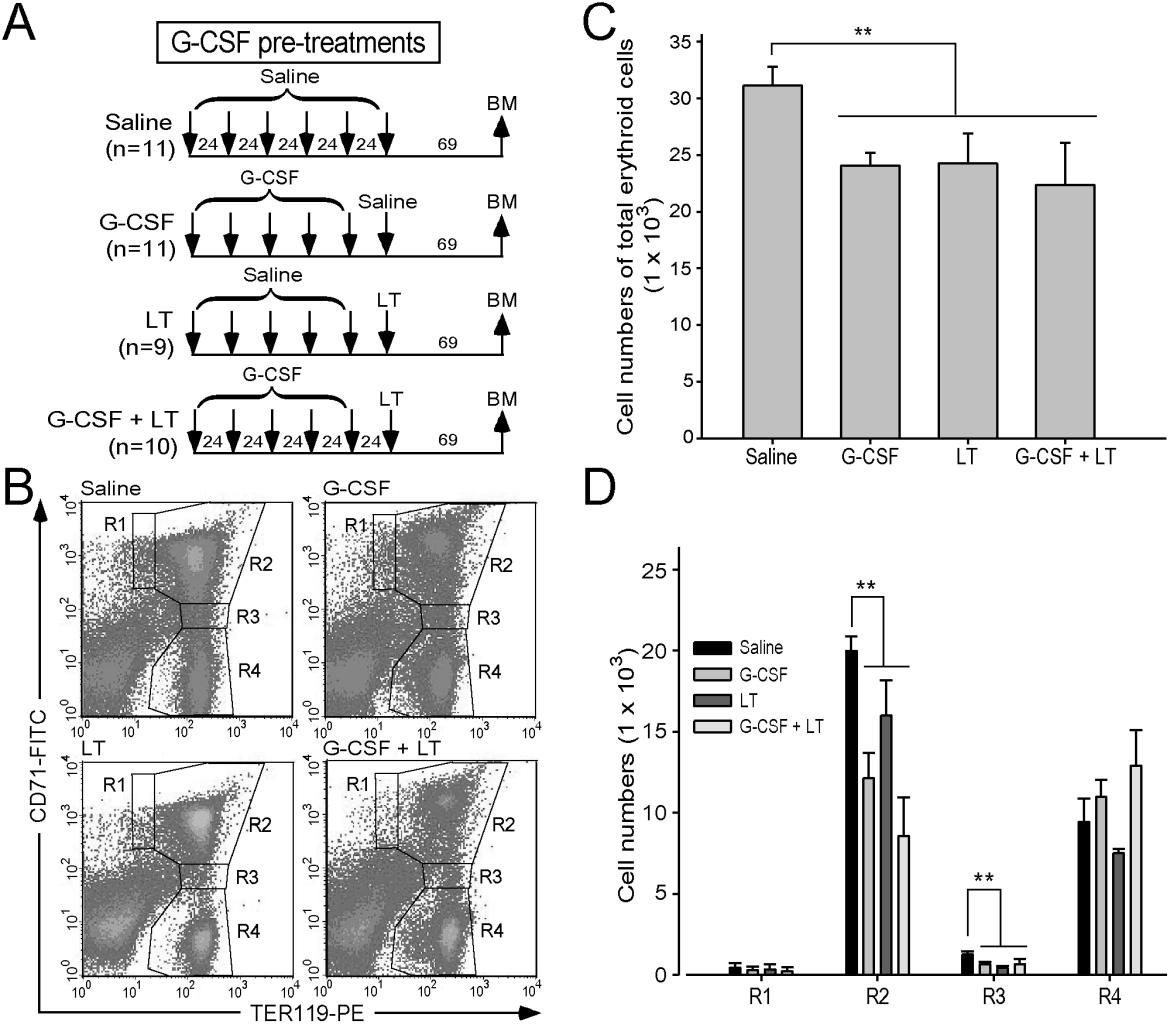
Regulation of G-CSF on bone marrow erythroblast populations. The experimental outlines are illustrated (A). Mice were treated with saline (n = 11), G-CSF (n = 11), LT (n = 9), or G-CSF plus LT (n = 10). Flow cytometry analysis analyzed erythroblast populations of BM cells at 69 h after LT challenges. The erythroblast cells were gated as R1 (CD71^high^, TER-119^med^), R2 (CD71^high^, TER-119^high^), R3 (CD71^med^, TER-119^high^), and R4 (CD71^low^, TER-119^high^) in all groups (B) as described [25]. The cell numbers of all erythroblast cells (sum of R1 to R4) (C) and individual erythroblast (R1, R2, R3, and R4) (D) in each group were quantified. **P<0.01 was compared between indicated groups. Data are showed as mean ± SD and represent the results from 2 independent experiments.

**Figure 3:**
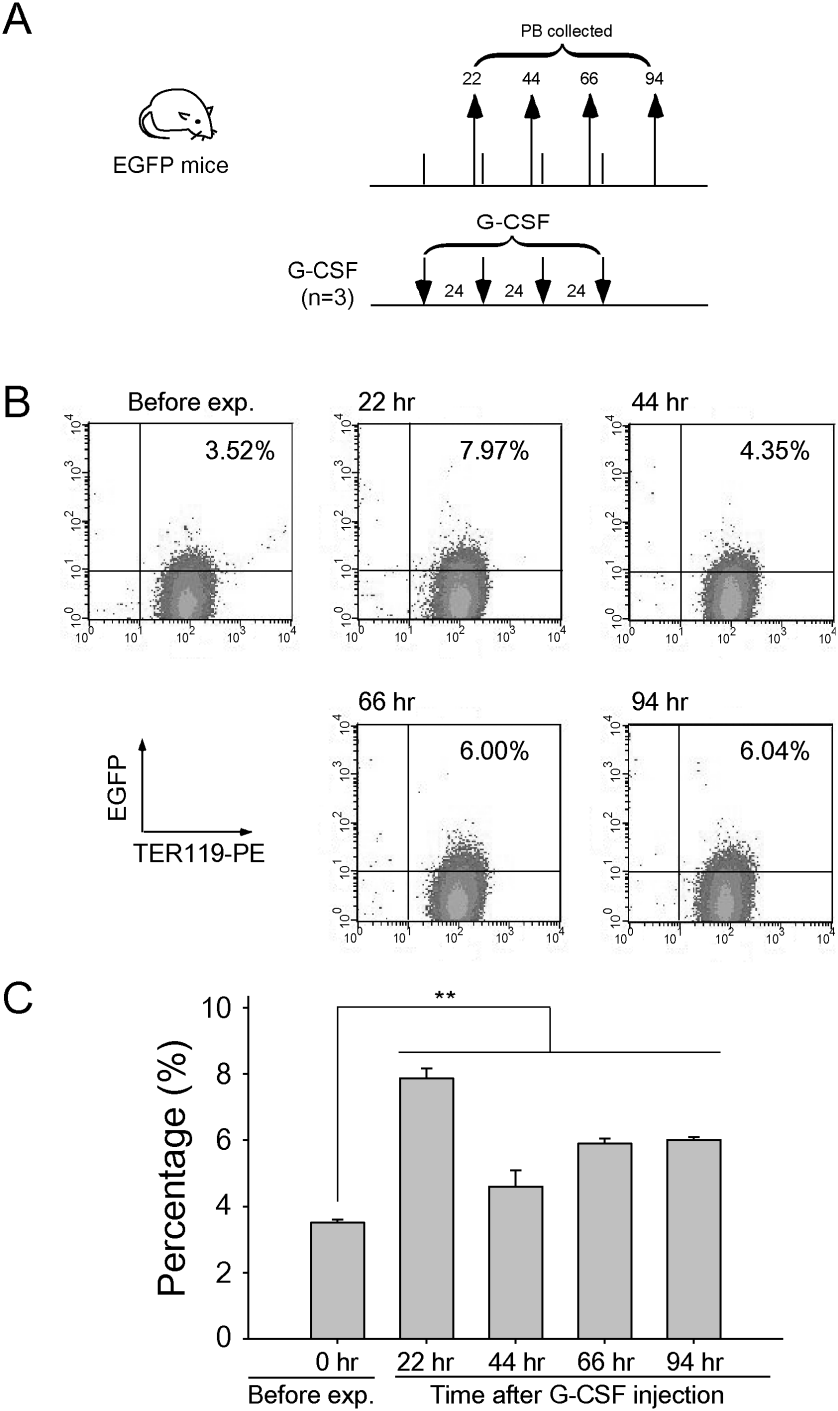
Mobilization of newly synthesized erythrocytes into peripheral blood by G-CSF. The experimental outline is illustrated (A). The EGFP mice were injected with G-CSF (n = 3). The percentage of EGFP^+^/TER119^+^ cells in peripheral blood (PB) was analyzed by flow cytometry (B) and quantified (C) at 22, 44, 66, and 94 h after G-CSF injection. PB collected from mice before G-CSF injection served as the negative control. **P<0.01 was compared to the negative control. Data are shown as mean ± SD.

After the promising analyses *in* *vitro* and *ex* *vivo*, this study investigated the erythropoietic progression *in* *vivo*. Our previous report revealed that LT suppressed erythropoiesis in bone marrow [15]. Following similar approaches [15], [25], we used surface expression of CD71 and TER-119 to verify the maturation status of various bone marrow erythroblasts under the G-CSF treatments with or without anthrax LT challenges. Although we found that G-CSF treatment rescued LT-induced erythrocytopenia (please see the following section), G-CSF pre-treatments could not overcome LT-mediated suppression on the cell numbers of both total erythroblast and individual subpopulations of erythroblast (R1 to R4 populations) (Figure 2C and 2D). This prompted us to verify whether G-CSF could mobilize mature erythrocytes into peripheral blood; we employed C57BL/6J mice with the whole-body-expressing enhanced-green-fluorescence-protein (EGFP) transgene. Prior to G-CSF analyses, we found that only a small fraction of EGFP^+^ RBCs was detectable in the peripheral blood of normal control groups (Figure 3, 3.5% cells, before exp. groups). This is likely because mature erythrocytes do not have a nucleus, and that newly differentiated RBCs, rather than aged RBCs, express detectable EGFP. We employed an acute hemorrhage model, in which 35% of total blood was removed, to provoke the natural induction of erythropoiesis to investigate whether newly synthesized erythrocytes contain additional fluorescence. Our data revealed that EGFP^+^/TER-119^+^ erythrocytes increased consistently by Days 2, 4, and 6 after acute anemia (Figure S2). This suggested that EGFP^+^/TER-119^+^ cells are newly synthesized erythrocytes. Using the same strategy, analyses revealed that G-CSF treatment can mobilize newly synthesized erythrocytes to peripheral blood in mice (Figure 3, beginning at 22 h after G-CSF injections).

### G-CSF treatment reduced LT-mediated mortality, erythrocytopenia, and thrombocytopenia

**Figure 4:**
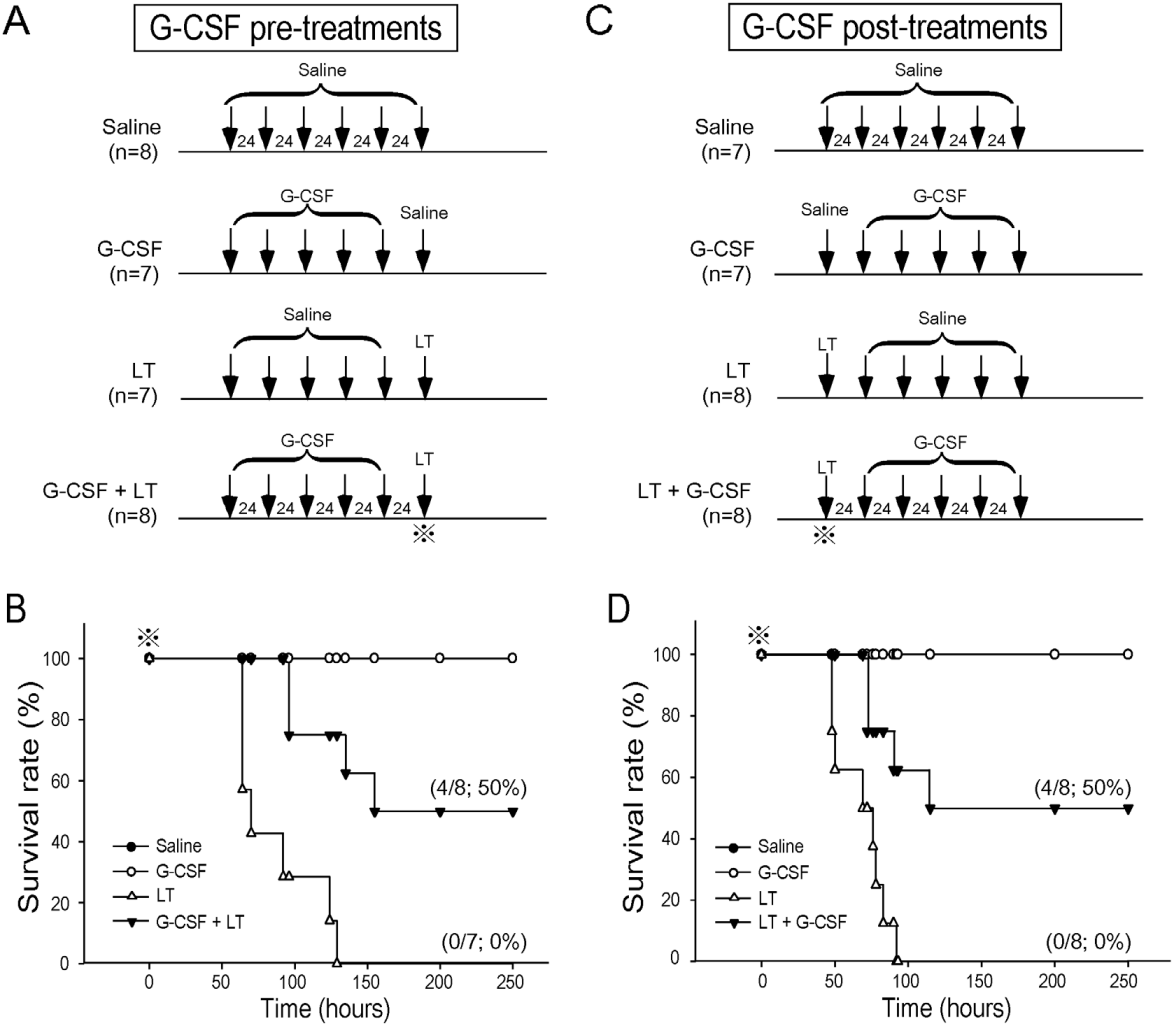
G-CSF treatments ameliorated LT-elicited mortality in mice. The experimental timetable (A), (C), and the survival rates of mice pre-treated (B) and post-treated (D) with G-CSF, LT, or G-CSF and LT are indicated. Saline treated mice served as negative controls. The symbol (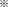) in (A) to (D) indicates the onset time point for recording survival rates.

**Figure 5:**
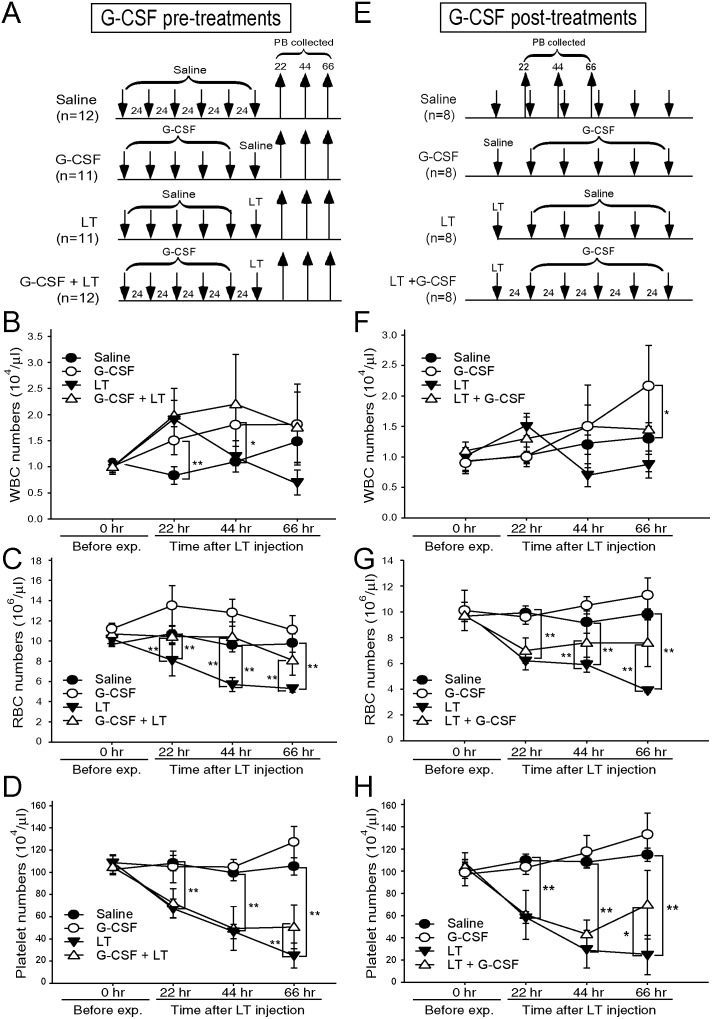
Amelioration of LT-induced erythrocytopenic response by G-CSF. The experimental outlines are indicated (A), (E). Mice were treated with saline (n = 12), G-CSF (n = 11), LT (n = 11), and G-CSF plus LT (n = 12) before (A) or saline (n = 8), G-CSF (n = 8), LT (n = 8), and LT plus G-CSF (n = 8) after (E) the LT challenges; their WBC, RBC, and platelet counts were subsequently analyzed at 22, 44, and 66 h after the LT challenges. Saline-treated mice were used as negative controls. *P<0.05, **P<0.01 were compared between the indicated groups. Data are shown as mean ± SD and represent the results from 2 independent experiments.

To investigate the ameliorative effect of G-CSF on LT, C57BL/6J mice were treated with G-CSF according to the manufacturer’s instructions (once daily for 5 d). Treatments of G-CSF were initiated 5 d before (Figure 4A) or 1 d after the challenges of a lethal dose of LT (Figure 4C). LT initiated mortality within 48 to 129 h (Figure 4B and 4D, LT groups). Administration of 5 doses of G-CSF before (Figure 4A) and after (Figure 4C) the LT challenges significantly improved survival rates (Figure 4B and 4D) (*P*<0.01). Treatments using saline, G-CSF, and LT alone served as the controls (Figure 4B and 4D). The peripheral white blood cell (WBC) counts of the G-CSF-treated groups increased approximately 2-fold at 22 and 44 h (Figure 5A and 5B), as well as at 66 h (Figure 5E and 5F) following the implementation of differing G-CSF regimens; this is in consistent with a previous G-CSF report [38]. Notably, both G-CSF treatments significantly ameliorated LT-induced erythrocytopenia (Figure 5C, G-CSF + LT vs. LT; Figure 5G, LT + G-CSF vs. LT). Compared with RBC counts, the ameliorative effect of G-CSF on LT-induced thrombocytopenia was somewhat later and was observed at approximately 66 h after LT treatment (Figure 5D and 5H). These results indicated that G-CSF positively regulated both RBC and platelet counts.

### G-CSF treatment induced erythrocytes to mobilize into peripheral blood faster than EPO

**Figure 6:**
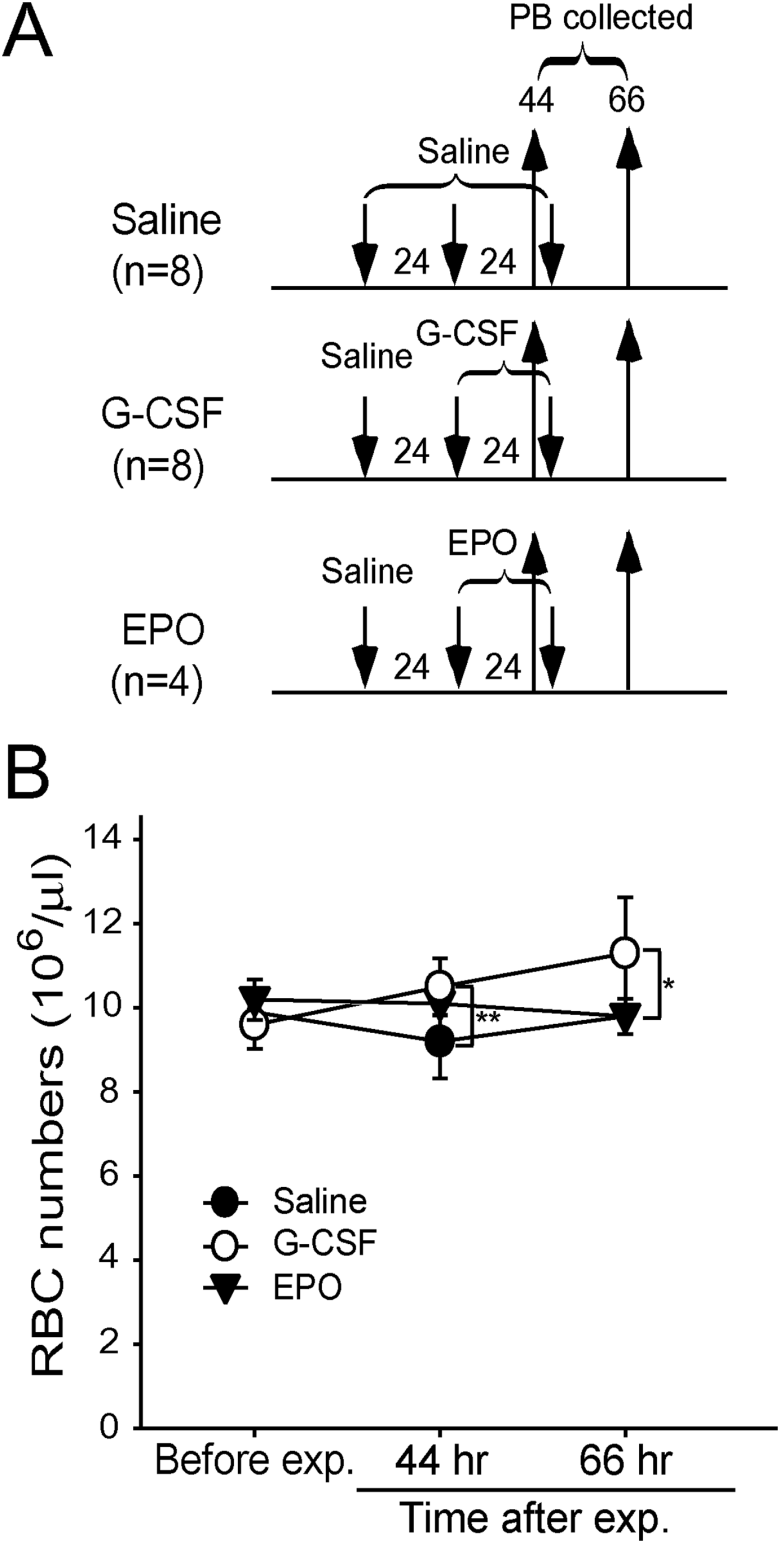
Fast mobilization of erythrocytes into peripheral blood by G-CSF versus EPO treatments. Experimental outline for measuring the PB-RBC counts of mice treated with G-CSF (n = 8) or EPO (n = 4) for 2 consecutive d (A). The PB RBC counts were measured before the experiments and at 44 and 66 h after the first saline injection (B). Data are shown as mean ± SD and represent the results from 2 independent experiments. Saline treated groups (n = 8) were used as the negative control. *P<0.05, **P<0.01 were compared to the negative control.

Our previous study showed that EPO up-regulated RBC counts in peripheral blood [15]. To compare the EPO and G-CSF treatments in their efficiency at increasing RBC counts, mice were injected with 2 doses of either G-CSF or EPO. The circulating RBC counts were measured (Figure 6). Our data revealed that G-CSF induced a faster increase of RBC counts, within 20 h of the first G-CSF administration, than the EPO treatment, in which no increased RBC counts were observed (Figure 6B, G-CSF vs. EPO). These results suggested that G-CSF induced a faster mobilization of erythrocytes into peripheral blood than that of EPO.

### Combined G-CSF and EPO had an ameliorative effect on anthrax-spore-induced mortality in C57BL/6J mice

**Figure 7:**
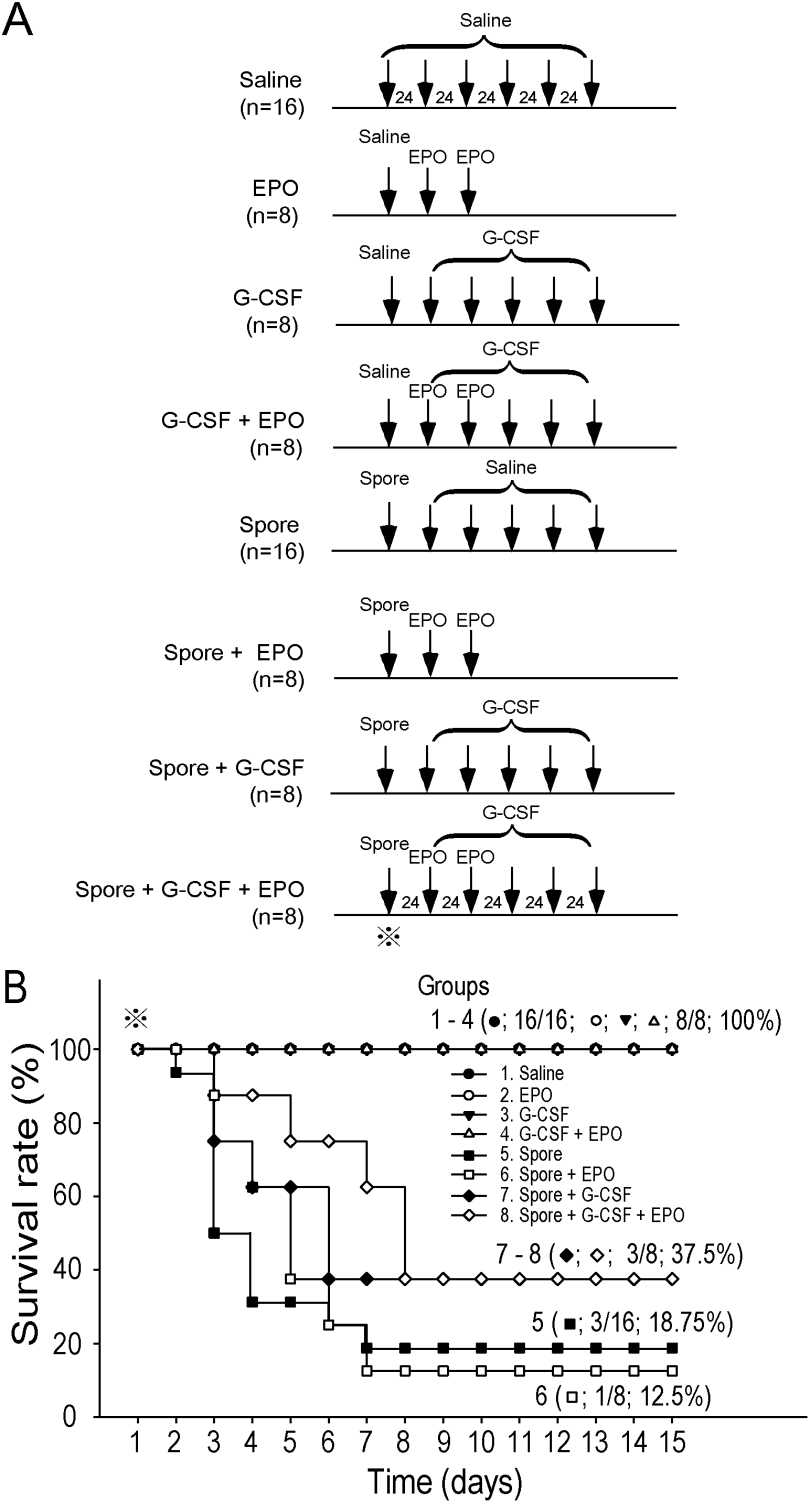
Post-treatments of G-CSF and EPO tended to ameliorate anthrax spore elicited-lethality in mice. Experimental outlines (A) and the survival rates of mice treated with G-CSF (n = 8), EPO (n = 8), and G-CSF combined with EPO (n = 8) after anthrax spore injection (B). Saline (n = 16), EPO (n = 8), G-CSF (n = 8), G-CSF and EPO (n = 8), and anthrax spore (n = 16) injected groups were used for comparisons. The symbol (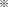) in (A) and (B) indicates the onset time point for recording survival rates. Data represent the results from 2 independent experiments.

Our previous report suggested that EPO ameliorates LT-mediated erythrocytopenia by enhancing erythropoiesis [15]. Because G-CSF increases the erythrocyte supply through a diverse mechanism by enhancing the mobilization of erythrocytes into peripheral blood, these results prompted us to investigate whether combined treatments using G-CSF and EPO may be more effective than respective single treatments alone. The analysis indicated that EPO treatment did not exert a protective effect on anthrax-spore-challenged mice (Figure 7B, Spore + EPO vs. Spore only). This is consistent with another line of evidence; the survival rates of anthrax LT-challenged mice increased only 25% following EPO post-treatment (Figure S3, LT + EPO vs. LT, *P* = 0.101). By contrast, G-CSF treatments with or without EPO effectively increased the survival rate of anthrax spore-challenged mice from 18.75% to 37.5% (Figure 7, Spore + G-CSF vs. Spore only). Post-treatments combining G-CSF and EPO prolonged the survival period of anthrax-spore-challenged mice (Figure 7, Spore + G-CSF + EPO vs. Spore + G-CSF). Statistical analysis revealed that the *P* value is marginally significant (Figure 7, *P* = 0.094, Spore + G-CSF + EPO vs. Spore; *P* = 0.088, Spore + G-CSF + EPO vs. Spore + EPO). These results suggested that combined treatment using G-CSF and EPO tended to ameliorate anthrax-spore-induced mortality in mice.

## Discussion

This study demonstrated that G-CSF, a stimulating factor for granulopoiesis, enhanced erythrocytic mobilization, by which it enhanced RBC counts in peripheral blood. This likely therefore rescued anthrax LT-induced anemia and mortality in mice.

Our *in* *vitro* (Figure 1A–1D) and *ex* *vivo* (Figure 1E–1H) evidence suggested that G-CSF may promote erythropoiesis. The *in* *vivo* analyses revealed that G-CSF ameliorated LT-induced erythrocytopenia in peripheral blood (Figure 5C and 5G), but did not increase erythroblast cell numbers in bone marrow (Figure 2). Therefore, the effects of G-CSF on erythropoiesis *in* *vivo* require clarification. One study demonstrated that G-CSF had a negative effect on bone marrow erythropoiesis in mice [39]. However, another study demonstrated that G-CSF treatments in humans increased immature reticulocytes in peripheral blood [40]. Clinical observations also found that the reticulocyte fraction, an assessment of immature erythroid cells in peripheral blood, was an early surrogate marker for the rise of CD34^+^ hematopoietic stem cells during G-CSF mobilization [41]. This evidence suggests that G-CSF is involved in regulating erythroid precursor cells in humans.

G-CSF induced fast mobilization of RBCs to peripheral blood within 20 h of the first G-CSF administration, compared with the EPO treatment (Figure 6). This data is consistent with the EGFP mice experiment, regarding the time in which G-CSF treatment increased the percentage of newly synthesized erythrocytes in peripheral blood (Figure 3). Thus, a single post-treatment dose of G-CSF was sufficient to save approximately 40% of mice within 24 h (Figure 4D, 100% and 60% survival rates in LT + G-CSF and LT, 48 h groups, respectively). The advantage of fast mobilization of RBCs of G-CSF can also be confirmed by the higher survival rate of G-CSF compared with EPO in LT-induced (Figure 4D vs. Figure S3B) and LT-spore-induced (Figure 7B) mortality in mice.

G-CSF is a multi-function cytokine that stimulates the proliferation and differentiation of myeloid precursors and modulates mature cells [29]. G-CSF is also used to prevent or shorten neutropenia in chemotherapy-induced or primary congenital neutropenia [42], and mobilize hematopoietic stem cells to peripheral blood for transplantation [43]. Probably because of the anti-apoptotic and anti-inflammatory effects, G-CSF has also been used to treat nonhematopoietic targets including cerebral ischemia [44], spinal cord ischemia [45], infarct heart [46], and end stage liver disease [47]. Consequently, the effects of G-CSF on other cell types (e.g., macrophage, lymphocyte, endothelial, dendritic cells, cardiomyocytes, and smooth muscle) may not be completely excluded from the rescue mechanism of G-CSF. Although the mechanism is unclear, combination therapy using G-CSF and EPO has been used to treat myelodysplastic anemia in clinical settings [32]–[36], [48]. This suggests that the medical community has empirically recognized the RBC-enhancing effect of the combination treatment using G-CSF and EPO. One critical aspect of G-CSF is the rapid induction of peripheral RBCs, a property superior to EPO, which may be applied to anthrax or other diseases with urgent RBC and oxygen demands. Further detailed and well-designed clinical studies are required to explore the therapeutic potential of combination treatment using G-CSF and EPO.

In this study, we demonstrated that G-CSF mobilized erythrocytes into peripheral blood. In addition, combined treatments of G-CSF and EPO tended to ameliorate anthrax spore-induced mortality. An optimized rescue protocol may provide new perspectives and assist the development of a feasible therapeutic strategy against anthrax.

## Supporting Information

Figure S1 **G-CSF treatments enhanced bone marrow erythroid colony numbers.** An *in* *vitro* erythroid colony-forming cell assay was performed using murine bone marrow (BM) cells incubated with [20 ng/ml (n = 6) or 764 ng/ml (n = 6)] or without G-CSF (n = 6). The erythroid colonies were confirmed by 3, 3′-diaminobenzidine tetrahydrochloride (DAB) staining (A) and quantified on Day 14 (B). Untreated BM cells were used as the control. **P<0.01 was compared to the untreated groups. Scale bar: 500 µm. Data are shown as mean ± SD.
(TIF)

Figure S2 **Mobilization of newly synthesized erythrocytes (EGFP^+^/TER-119^+^) into peripheral blood by acute anemia in EGFP transgenic mice.** Experimental timetable used in acute anima assay (A). During acute anima (after aspirating 35% of total blood), the population of EGFP^+^/TER-119^+^ cells in peripheral blood (PB) of EGFP mice was gated as R1 and quantified. The total cell number was defined as 100%. The percentage of R1 was analyzed by flow cytometry (B) on Day 2, 4, and 6 after the removal of 35% of total blood. PB samples from mice before acute anemia were used as negative controls. Data were collected from 2 representative EGFP mice.
(TIF)

Figure S3
**Post-treatments of EPO increased the survival rates of LT-challenged mice.** Experimental timetable (A). The survival rates of mice challenged with EPO (n = 4), LT (n = 4), or LT plus EPO (n = 4) are shown (B). Saline-treated mice were used as controls (n = 4). C57BL/6J mice were retro-orbitally injected with recombinant human EPO (rhEPO, Neorecormon, Roche, Mannheim, Germany) (2 IU/g, in 250 µl saline) twice every 24 h after the challenges of a lethal dose of LT (1.5 mg/kg in 250 µl saline, retro-orbitally injected). The symbol (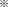) in (A) and (B) indicates the onset time point for recording the survival rates of mice.(TIF)

Methods S1 **Supplemental experimental procedures.**
(DOC)
