# Supplementary figures and images for: Erythrocytic Mobilization Enhanced by the Granulocyte Colony-Stimulating Factor Is Associated with Reduced Anthrax-Lethal-Toxin-Induced Mortality in Mice
Source: PLoS One. 2014 Nov 10;9(11):e111149. doi: 10.1371/journal.pone.0111149 (PMC4226491; doi:10.1371/journal.pone.0111149)

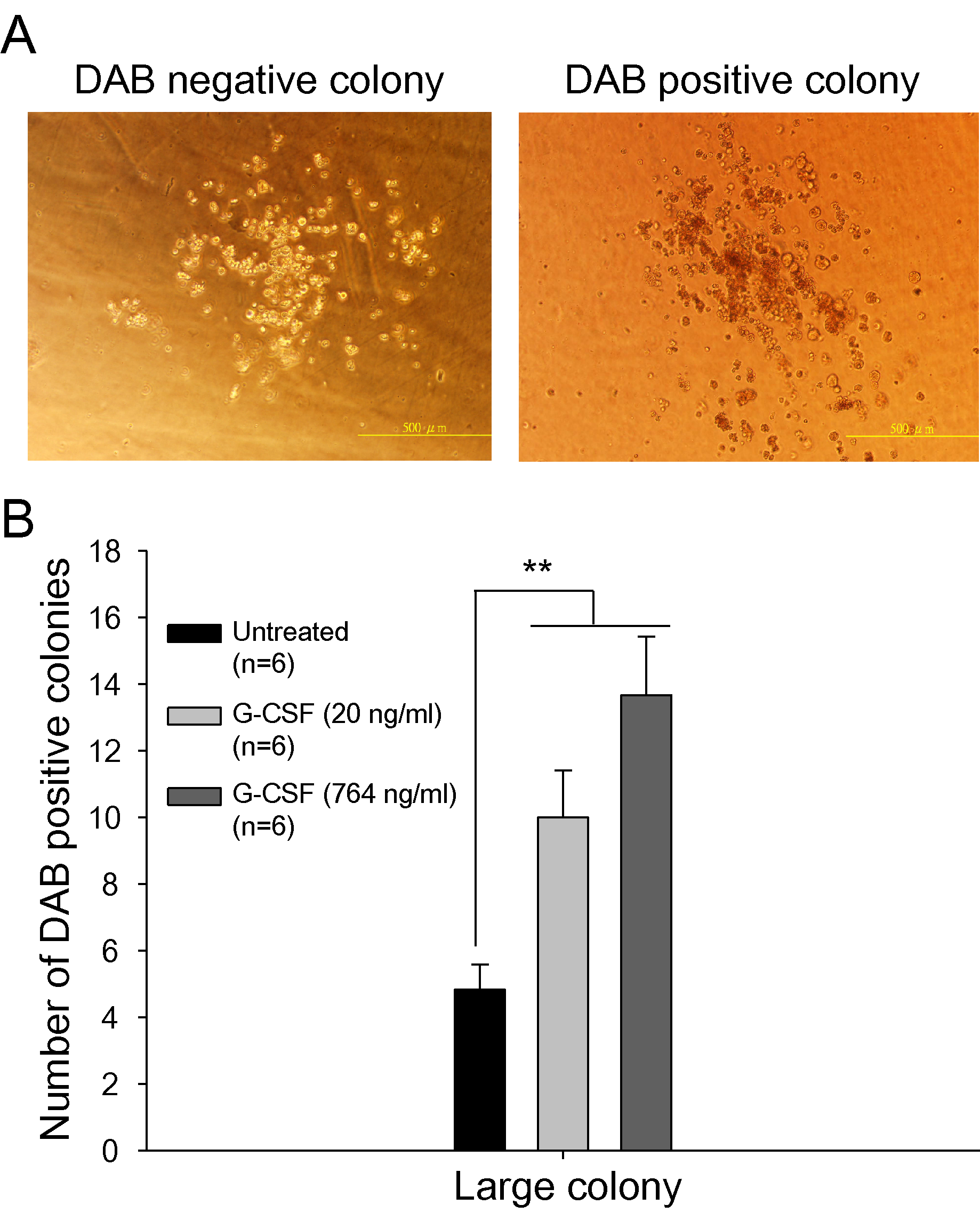

Supplement: Figure S1 — G-CSF treatments enhanced bone marrow erythroid colony numbers. An in vitro erythroid colony-forming cell assay was performed using murine bone marrow (BM) cells incubated with [20 ng/ml (n = 6) or 764 ng/ml (n = 6)] or without G-CSF (n = 6). The erythroid colonies were confirmed by 3, 3′-diaminobenzidine tetrahydrochloride (DAB) staining (A) and quantified on Day 14 (B). Untreated BM cells were used as the control. **P<0.01 was compared to the untreated groups. Scale bar: 500 µm. Data are shown as mean ± SD. (TIF) [file pone.0111149.s001.tif]

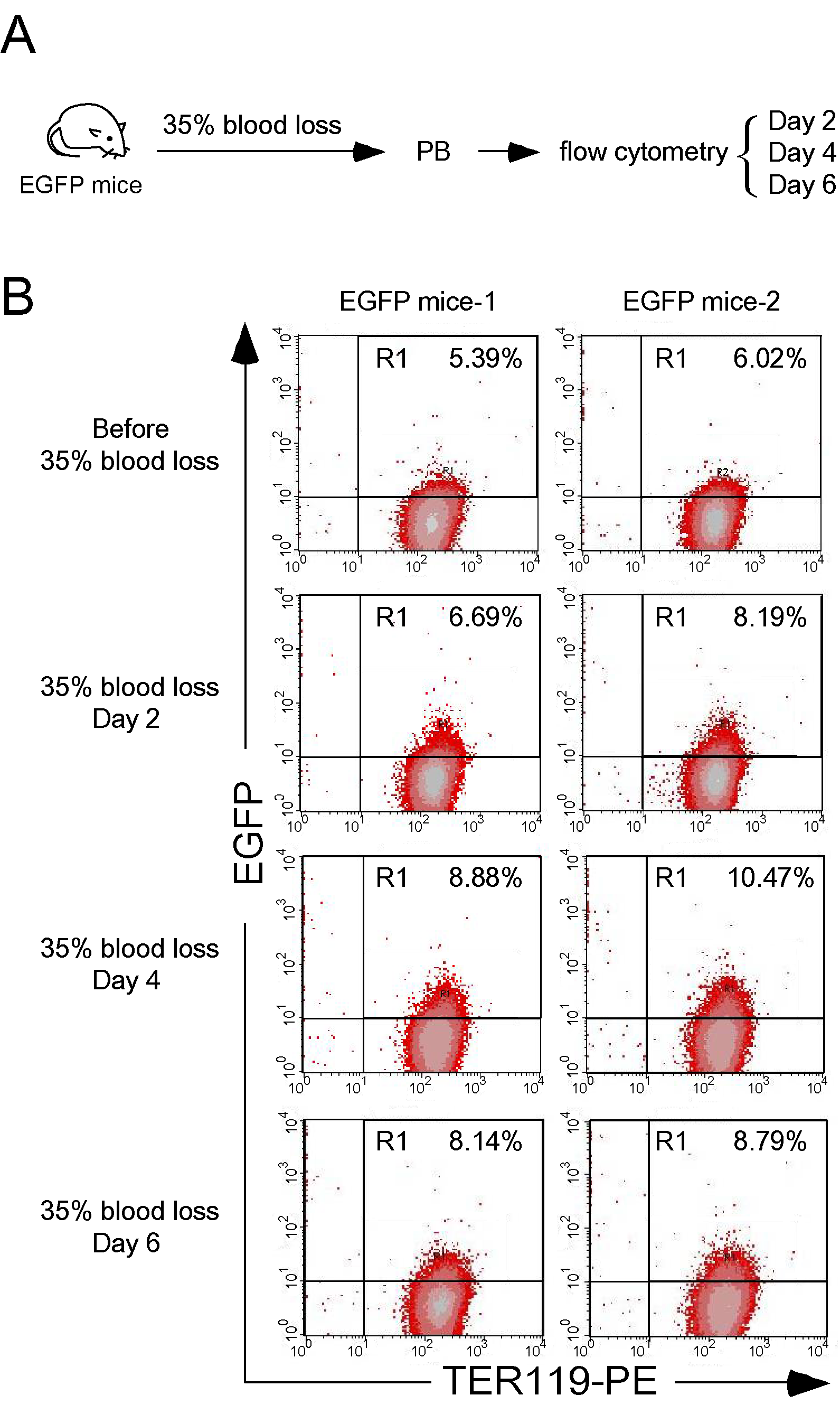

Supplement: Figure S2 — Mobilization of newly synthesized erythrocytes (EGFP+/TER-119+) into peripheral blood by acute anemia in EGFP transgenic mice. Experimental timetable used in acute anima assay (A). During acute anima (after aspirating 35% of total blood), the population of EGFP+/TER-119+ cells in peripheral blood (PB) of EGFP mice was gated as R1 and quantified. The total cell number was defined as 100%. The percentage of R1 was analyzed by flow cytometry (B) on Day 2, 4, and 6 after the removal of 35% of total blood. PB samples from mice before acute anemia were used as negative controls. Data were collected from 2 representative EGFP mice. (TIF) [file pone.0111149.s002.tif]
