# Supplementary material for: Erythrocytic Mobilization Enhanced by the Granulocyte Colony-Stimulating Factor Is Associated with Reduced Anthrax-Lethal-Toxin-Induced Mortality in Mice
Source: PLoS One. 2014 Nov 10;9(11):e111149. doi: 10.1371/journal.pone.0111149 (PMC4226491; doi:10.1371/journal.pone.0111149)
Supplement: Methods S1 — Supplemental experimental procedures. (DOC) [file pone.0111149.s004.doc]

**Methods S1**

**DAB staining**

3, 3`-diaminobenzidine tetrahydrochloride (DAB, Sigma) staining was performed as described with slight modification to identify the erythroid colonies in erythroid colony-forming cell assay. One DAB tablet was dissolved in 15 ml of 0.05 M Tris-HCl buffer, pH 7.6. 40 μl of 30% hydrogen peroxide solution were added to 2 ml of DAB solution prior to use, and 500 μl of DAB solution was layered over the dishes and incubated at room temperature for 12 h. The brown colonies were counted and the images were taken under inverted microscope (OLYMPUS Inverted Microscope IX71).

**Acute blood loss in EGFP mice**

EGFP mice [C57BL/6J-Tg (Pgk1-EGFP) 03Narl, males, 6-8 wk of age] were obtained from the National Laboratory Animal Center (Taipei, Taiwan) and kept in pathogen-free environment in the experimental animal center of Tzu Chi University. Acute blood loss was performed by aspirating 35% of total blood (approximately 656 μl/25 g mouse) from the retro-orbital plexus of EGFP mice. To detect erythrocytes specific surface marker, 50 μl of retro-orbital blood samples were collected on Day 2, 4, and 6 after acute blood loss, and then mixed with 450 μl anticoagulant acid citrate dextrose, formula A, ACD-A (1:9). Cells were incubated with 3 μl of R-Phycoerythrin (R-PE)-conjugated rat anti-mouse TER-119 antibody (BD Immunocytometry System) in 300 μl RPMI-1640 medium at 37°C for 1 h and then analyzed using a FACSCalibur flow cytometer and the CellQuest^TM^ Pro program.

**Post-treatments of EPO enhanced survival rate of LT-challenged mice**

C57BL/6J mice (males, 8-10 wk of age) were retro-orbitally challenged with recombinant human EPO (rhEPO, Neorecormon ®, Roche, Mannheim, Germany) (2 IU/g, in 250 μl saline) twice at 24 and 48 h after injection of a lethal dose of LT (1.5 mg/kg in 250 μl saline, retro-orbital injection). Experimental groups injected with saline or EPO alone served as controls. The survival time and mortality of mice were recorded after the LT challenge. All surviving mice were monitored each day for 2 subsequent mo.

**Reference**

1. Ogawa M, Parmley RT, Bank HL, Spicer SS (1976) Human marrow erythropoiesis in culture. I. Characterization of methylcellulose colony assay. Blood 48: 407-417.
